# A Rare Case of an Exploratory Laparotomy to Treat a Liver Abscess Secondary to Foreign Body Ingestion

**DOI:** 10.7759/cureus.25747

**Published:** 2022-06-08

**Authors:** Martha Chavez, Srijesa Khasnabish, Ian Landry, Merjona Saliaj

**Affiliations:** 1 Internal Medicine, St. George's University School of Medicine, New York, USA; 2 Internal Medicine, New York Institute of Technology College of Osteopathic Medicine, New York, USA; 3 Medicine, Icahn School of Medicine at Mount Sinai (NYC Health and Hospitals: Queens), New York, USA; 4 Medicine, Icahn School of Medicine at Mount Sinai at Queens Hospital Center, New York, USA

**Keywords:** indications for surgery, foreign bodies/surgery, liver abscess drainage, foreign body removal, ingested foreign body

## Abstract

A 49-year-old female presented to the hospital with complaints of generalized weakness, subjective fevers, and chills. In the emergency department (ED), she was found to be hypotensive and tachycardic and met the sepsis criteria. A CT scan of the abdomen and pelvis (CT A/P) with contrast revealed a liver abscess and a foreign body (FB) that was suspected to be the cause of the liver abscess. Of note, the patient had undergone a recent dental procedure due to an infected root canal, which had involved a dental screw. The patient was uncertain whether the dental screw had been removed, but she felt as though it was no longer there. At this time, the clinical suspicion was high for FB secondary to this dental procedure. The patient underwent interventional radiology (IR)-guided liver abscess drainage and magnetic resonance cholangiopancreatography (MRCP) for the evaluation of the FB. An esophagogastroduodenoscopy (EGD) was performed, but no evidence of the FB was found. This warranted an exploratory laparotomy (EL) to ensure the successful removal of the FB. Upon gross visualization by surgery, the FB was revealed to be a bone that the patient did not recall ingesting. However, surgical pathology evaluation revealed that the FB was actually a plastic stick. This rare case highlights the clinical approach to FB ingestion when complicated by liver abscess, as well as successful treatment with EL as opposed to laparoscopy which is the procedure of choice.

## Introduction

Foreign body (FB) ingestion is rare in the adult population. Ingestion is often accidental or occurs in individuals with psychiatric disorders, developmental delay, alcohol intoxication, or those who are incarcerated as a means of obtaining release to a treatment facility [1]. According to the American Society for Gastrointestinal Endoscopy, FB at the level of the esophagus will often pass spontaneously within 24 hours [2]. However, with sharp objects such as animal bones, there is an increased risk of perforation of the esophagus or gastrointestinal tract (GIT). Sharp objects, in particular, can be missed on plain radiographs, and hence imaging with contrast is often necessary. Non-invasive evaluation with endoscopy is recommended prior to surgical removal, particularly if the object is localized above the duodenum [3]. The failure to remove the object endoscopically and a worsening clinical picture suggestive of peritonitis or sepsis warrant laparoscopic intervention or laparotomy for successful removal of the FB [2].

We report the case of a 49-year-old woman who suffered a liver abscess and subsequent sepsis secondary to accidental ingestion of a plastic stick.

## Case presentation

A 49-year-old Guyanese female with hypertension, hyperlipidemia, gastroesophageal reflux disease, and trigeminal neuralgia presented to the emergency department (ED) with fevers, chills, and pain secondary to a decayed tooth in the right lower jaw for one week. She reported undergoing a dental procedure one week ago to address an infected root canal with subsequent dental screw manipulation but had not been prescribed any antibiotics. Upon arrival to the ED, she denied chest pain, shortness of breath, drooling, or difficulty swallowing. She was hypertensive, but afebrile. She was discharged after empiric treatment for a dental infection with amoxicillin 500 mg by mouth every eight hours for seven days and her pain was well controlled with oral ibuprofen 600 mg.

Nine days later, she returned to the ED complaining of worsening fever, chills, and new-onset intermittent diarrhea that was dark-colored and foul-smelling and associated with generalized abdominal pain. She had a blood pressure of 74/55 mmHg, pulse rate of 108 beats per minute, temperature of 97.7 °F, respiratory rate of 19 breaths per minute, and pulse oximetry of 98% while breathing ambient air. Laboratory values on admission and discharge are listed in Table 1. Of note, she presented with leukocytosis and elevated alkaline phosphatase and aspartate and alanine transaminases.

**Table 1:** Lab values on admission and discharge

Variables	On admission (early April)	On discharge (late April)	Unit	Reference range
White blood cells	23.13	7.24	units/mc	4.8–10.8
Neutrophils	82.10	63.80	%	44–70
Total bilirubin	1.0	0.4	mg/dL	0.0–1.2
Alkaline phosphatase	126	37	U/L	35–104
Aspartate transaminase (AST)	58	41	U/L	5–32
Alanine transaminase (ALT)	42	19	U/L	0–33

**Figure 1:**
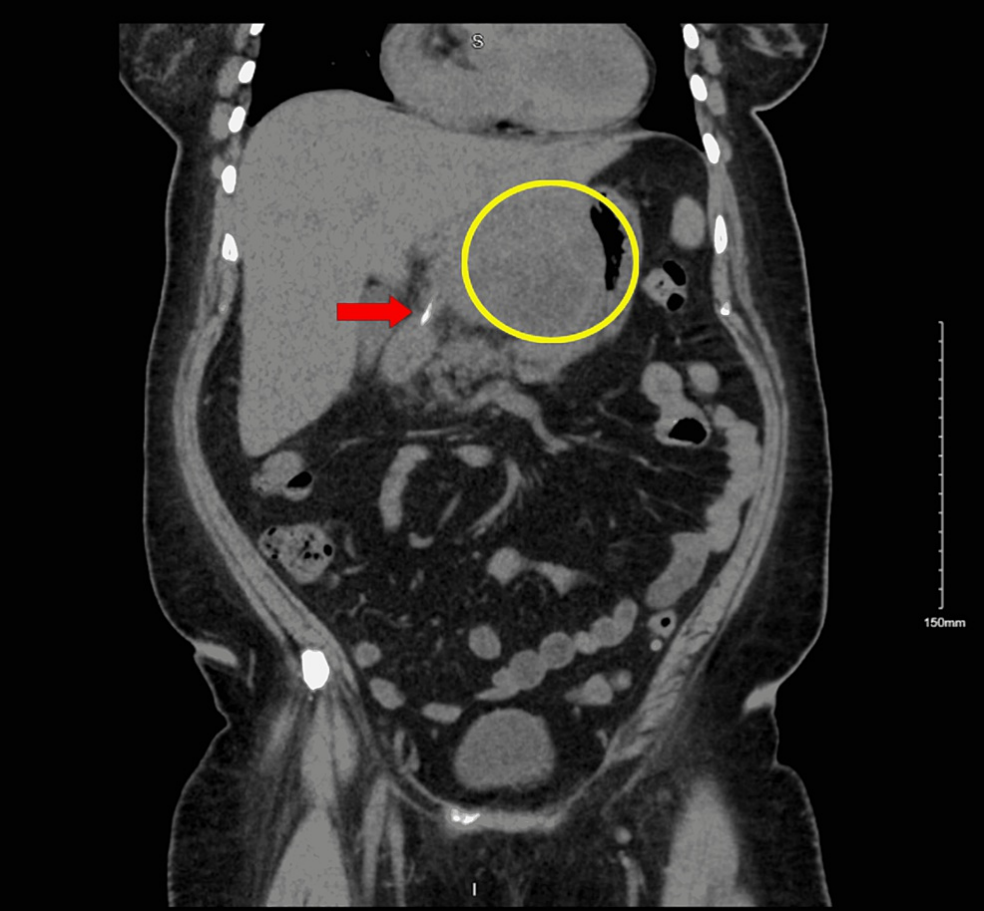
Initial CT of the abdomen and pelvis with contrast, coronal view Impressions: 1) There is an 8 x 8 x 10 cm irregular enhancing hypodense lesion occupying a large portion of the left hepatic lobe. Multiple gas pockets are also present. This finding is concerning for intrahepatic abscesses. 2) 3-mm linear hyperdensity in the region of the duodenal bulb/pylorus that appears to extend through the superior wall into the left hepatic lobe. This may be an ingested foreign body with perforation, causing the intrahepatic abscess. 3) Layering debris in the gallbladder suggestive of stones/sludge CT: computed tomography

Initial CT of the abdomen and pelvis (CT A/P) with contrast revealed a left intrahepatic abscess and a 3-mm linear hyperdensity from the duodenal bulb extending through the superior aspect of the left hepatic lobe. The patient denied any memory of ingesting animal bones. Also, the patient had undergone a recent dental procedure involving a dental screw and was uncertain regarding the presence of the screw. Because of this history, the linear hyperdensity finding on CT A/P with contrast raised suspicion regarding the presence of an FB (Figure 1). Two days later, the patient underwent interventional radiology (IR)-guided liver abscess drainage with Jackson-Pratt (JP) surgical drain placement and was placed on intravenous infusion of vancomycin 1250 mg every 12 hours, Flagyl 500 mg every eight hours, and Zosyn 3.375 g every six hours.

**Figure 2:**
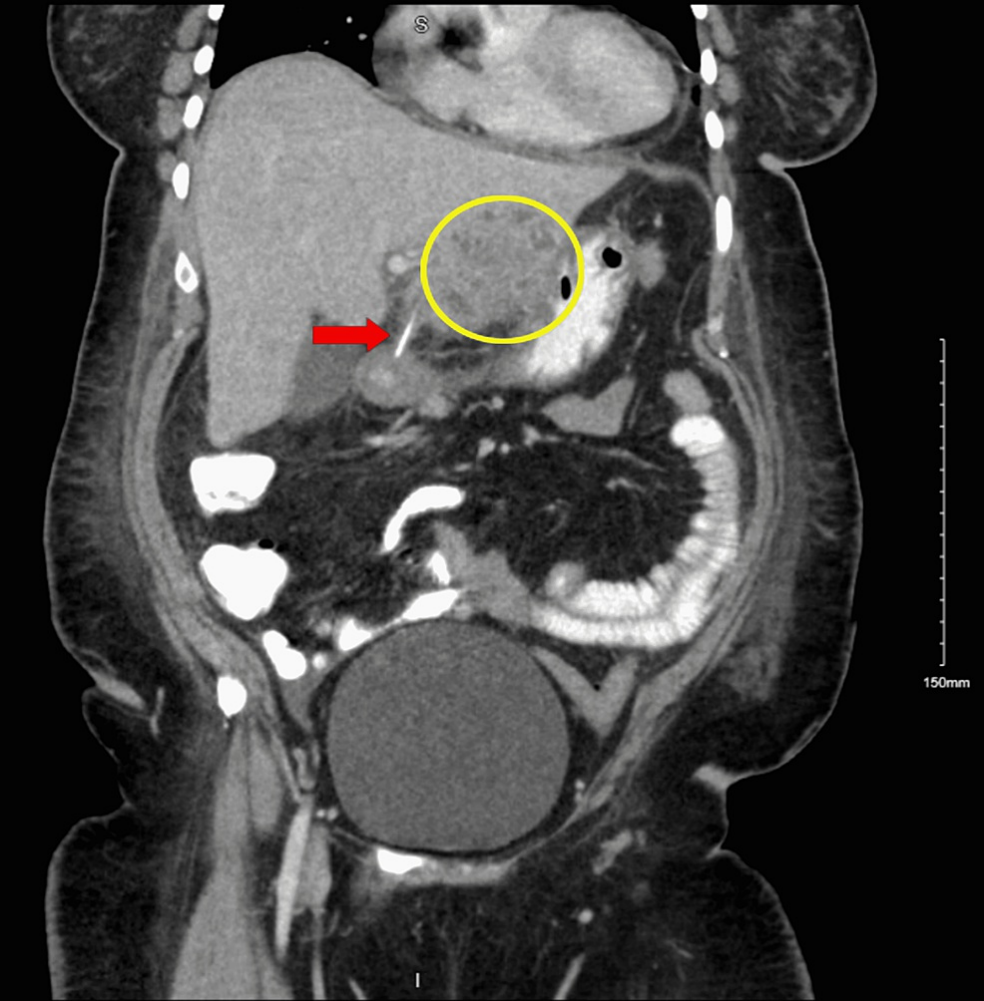
Repeat CT of the abdomen and pelvis with contrast (six days after the initial CT), coronal view Impression: 1) There has been interval placement of a JP surgical drain within the previously noted hepatic abscess with a significant interval decrease in size now measuring 3.6 cm (previously 10 cm). 2) There is a stable 3-mm linear hyperdensity noted in the region of the duodenal bulb extending through the superior wall into the left hepatic lobe concerning for ingested FB. There is an enlarged periaortic lymph node noted at the diaphragmatic hiatus measuring approximately 1.6 cm. Findings may be reactive CT: computed tomography

Initially, her drainage output was consistent with copious, thick secretions, which were ultimately found positive for Haemophilus parainfluenzae and Streptococcus intermedius. After eight days, her drainage output ceased and her tubes were removed. A magnetic resonance cholangiopancreatography (MRCP) was performed to evaluate for biliary involvement, which confirmed the presence of an FB in the proximal duodenum. A subsequent esophagogastroduodenoscopy (EGD) was performed but it failed to visualize the FB within the lumen (see Table 2 for the timeline). Repeat CT A/P following these procedures showed that the liver abscess had decreased in size, but there was no change in the position of the FB (Figure 2). 

Upon surgical consultation, the patient was taken to the operating room for an exploratory laparotomy (EL), repair of left lobe laceration, removal of the FB from hepatic-duodenal ligament, and washout of liver abscess with JP drain placement. Following the EL, her abdominal pain and fever resolved. Upon gross examination, surgery believed the FB to be a bone, but upon surgical pathology evaluation, the FB was revealed to be a part of a tan plastic stick measuring 3.1 cm in length and 0.1 cm in diameter. The infectious disease team was consulted and they recommended Augmentin 875-125 mg by mouth every 12 hours for 26 days upon discharge. They also recommended a repeat CT A/P as an outpatient in one week. The patient was discharged six days after the EL and after the removal of the JP drain. Table 1 lays out the timeline of the procedures, and Figure 2 illustrates the repeat CT performed after MRCP and EGD.

**Table 2:** Procedure timeline

Procedure	Number of days since admission	Findings
Interventional radiology-guided liver abscess drainage	2 days	Body guild culture revealed a few gram-positive cocci in pairs, rare Haemophilus parainfluenzae, and a few Streptococcus intermedius. Jackson-Pratt surgical drain 8 days after the procedure
Magnetic resonance cholangiopancreatography (MRCP)	4 days	Confirmed 1) hepatic abscess and 2) foreign body in the first portion of the duodenum
Esophagogastroduodenoscopy (EGD)	5 days	Found no evidence of foreign body
Exploratory laparotomy	12 days	The foreign body was removed and it was thought to be a bone upon gross visualization by surgery

## Discussion

Accidental FB ingestion is a common clinical problem. The FBs most commonly ingested by adults are fish bones and chicken bones. In about 80% of cases, the ingested material passes through the GIT uneventfully and requires no further intervention. However, in about 20% of cases, an endoscopy is indicated, and in 1% of cases, surgery needs to be performed [4]. The clinical approach depends on the type of material ingested and the patient’s symptoms and physical findings. Therefore, endoscopic or surgical intervention is indicated if significant symptoms develop or if the object fails to progress through the GIT [3].

A rare consequence that may result when the FB does not progress through the GIT is the formation of a liver abscess. A liver abscess is defined as a pus-filled mass that can develop from an injury to the liver or from an intra-abdominal infection disseminated from the portal vein [5]. About 50% of liver abscesses occur in the right lobe of the liver as there is more blood supply in this area [5]. The less common locations are the left lobe and caudate lobe of the liver [5]. As the FB passes through the GIT, there are many locations where it can lodge and thus perforate. Possible sites include the pylorus, duodenum, duodenojejunal junction, and ileocecal region [6]. In this case, CT showed a linear hyperdensity in the region of the duodenal bulb and pylorus that extended into the left hepatic lobe (Figures 1, 2). This finding implied possible migration and penetration of the FB, leading to hepatic abscess formation.

Although the incidence of liver abscesses is low, early detection and management are essential due to the significant mortality risk in untreated patients [5]. In most cases, the ingested FB passes through the GIT and is excreted in the stool within one week [6]. However, patients who do not excrete the FB uneventfully present with symptoms such as epigastric or abdominal pain, fever, chills, anorexia, fatigue, vomiting, nausea, and weight loss [7]. If the FB does not pass on its own, curative endoscopic or laparoscopic surgery is warranted, as seen in this patient [3].

Patients suspected of FB ingestion undergo CT imaging to define the nature, location, and potential complications related to the object. Objects below the cricopharyngeus are retrieved via flexible endoscopy. When objects are not within safe reach and unable to be extracted with flexible endoscopy, surgery is indicated [8]. Historically, EL has been the mainstay of treatment for patients requiring surgery. However, laparoscopic removal is an appealing alternative because it is less invasive, less painful, and offers a faster recovery [9]. Laparoscopic surgery also yields better outcomes than laparotomy in terms of the duration of surgery and intraoperative blood loss [10]. Hence, it is the procedure of choice in failed endoscopic removals [9].

In this case, EL was the procedure of choice as opposed to laparoscopic surgery given that the patient had a persistent and clearly symptomatic left liver abscess associated with an FB in the hepatoduodenal ligament. FB ingestion is a common occurrence, and although few cases require open surgical intervention, urgent surgical removal is indicated in cases of persistent inflammation leading to unremitting symptoms as was seen in this case [11]. EL is rarely required for ingested FB removal.

## Conclusions

An ingested FB usually passes through the GIT successfully and is excreted without complications. However, complications such as a liver abscess can occur when the FB is lodged in the GIT instead. Although the incidence of this complication is rare, it results in significant symptoms and mortality risk for the patients. In order to manage a patient with complications due to FB ingestion, modalities such as CT imaging, EGD, and surgery may be required. When the FB is unable to be extracted using EGD, surgery is then performed. Laparoscopic surgery is the procedure of choice as it is less invasive and provides faster recovery; however, in this case, an EL was performed due to persistent and symptomatic abscess formation.
